# Hormonal Contraception and Violent Death: The Physiological and Psychological Links

**DOI:** 10.3389/fnbeh.2021.667563

**Published:** 2021-07-30

**Authors:** Angela Lanfranchi

**Affiliations:** ^1^Department of Surgery, Rutgers-Robert Wood Johnson Medical School, New Brunswick, NJ, United States; ^2^Breast Cancer Prevention Institute, Whitehouse Station, NJ, United States

**Keywords:** hormonal contraception, violent death, intimate partner violence, major histocompatibility genes, human vomeronasal organ, mate retention behaviors, pheromones, brain structure

## Abstract

In the past decade, two large prospective cohort studies of British and American women have been conducted which found a statistically significant increase in the risk of violent death in ever-users of hormonal contraceptives. Research on the effects of hormonal contraceptives upon the behaviors of intimate partners and on the physiology of women using hormonal contraceptives has provided insight into the possible basis for the resulting increase in violent death. This review examines the changes that are potential contributors to the reported increase.

## Introduction

In 2010, the British Medical Journal (BMJ) published updated cohort evidence regarding overall mortality from the Royal College of General Practitioners’ (RCGP) Oral Contraception Study (Hannaford et al., 2010). The study concluded that oral contraceptives were not associated with an overall increased long-term rate of death among ever-users. However, as stated by the authors, “We are unable to explain the increased risk of violent and accidental deaths in our study among ever-users of oral contraceptives. The persistence of this effect is noteworthy.” (Hannaford et al., 2010). They found a statistically significant increased risk of 92% in ever-users and a statistically significant increase of 116% if the contraceptives were used longer than 8 years. This increased risk associated with an increased duration of use suggested a biological underpinning of the finding. Approximately one-third of the violent deaths were suicides (Hannaford et al., 2010). The study also confirmed the 1999 Beral study’s earlier finding (Beral et al., 1999) in the same RCGP cohort of a statistically significant increased risk in violent death.

In response to the study by a letter to the editor, Roberts (2010) wrote: “There is evidence that use of oral contraception alters women’s baseline preferences for men such that pill users prefer men who are relatively similar to themselves at loci in the major histocompatibility complex (MHC). One consequence of being partnered with relatively MHC-similar men is that such women express lower sexual responsivity toward their long-term partner compared with women in relatively MHC-dissimilar couples, reject sexual advances from their partner more frequently, and report having had more extra-pair partners. Other evidence points to MHC- similar couples being more likely to experience problems conceiving children and having less healthy children due to lower MHC-heterozygosity. Cumulatively, these effects could have a real impact on the quality of spousal relationships. It is not unreasonable to suspect that such effects could also influence rates of intimate partner violence. This is the most common cause of nonfatal injury among women (Kyriacou et al., 1999) and accounts for more than a third of women murdered in the US (Kellermann and Mercy, 1992)”. Roberts (2010) described ways in which hormonal contraceptives could lead to female behaviors that may potentially cause conflict, which, as a result, could induce intimate partner violence leading to death. Although references were cited in this response and “common sense” might cause this opinion to be formed, clearly more data would be needed before the risk of violent death as a result of oral contraceptives be used to form a clinical opinion to be used while caring for patients.

In the United States, intimate partner violence (IPV) has become all too common as evidenced by conservative estimates which indicate that 20–30% of women in the United States have experienced IPV in their lifetime (Chang, 2014). The American College of Family Practitioners and American College of Obstetrics and Gynecology recommend routine IPV screening of women (American College of Obstetricians and Gynecologists, 2012; Dicola and Spaar, 2016). Thirty percent of female homicide victims from 1976–2005 were killed by intimate partners according to the U.S. Department of Justice (Catalano, 2007). IPV is also the leading cause of death of pregnant women in several U.S. states and Finland (Gissler and Hemminki, 1999; Maryland Department of Health and Mental Hygiene, 2013; Wallace et al., 2020).

In 2014, a 36-year follow-up of the Nurses’ Health Study (Charlton et al., 2014) was also published in the BMJ which confirmed previously reported findings (Hannaford et al., 2010), in a prospective cohort of American nurses. Although the all-cause mortality data did not significantly differ between ever and never-users of oral contraceptives, they did note increased rates of violent or accidental deaths. The results indicated a 20% statistically significant increase in the risk of violence and/or accidental deaths in ever-users. The authors reported that “However, violent or accidental deaths were more common among ever users (hazard ratio 1.20, 95% confidence interval 1.04–1.37), which was driven by suicide (1.41, 1.05–1.87) compared with non-suicide violent or accidental deaths (1.13, 0.97–1.32).” (Charlton et al., 2014). However, there was no data showing a dose-response effect in the duration of use, unlike the RCGP cohort. They concluded, “However, it is unlikely this is a causal association given the lack of a biological mechanism as well as inconsistent results in our other analyses, such as duration of oral contraceptive use with violent death.” (Charlton et al., 2014).

This review will examine data published concerning the physiology resulting from women’s hormonal contraceptive use and their impact on the psychology of both women and their mates in heterosexual relationships. Specifically, there is evidence for a human vomeronasal organ which can mediate the choice preference for intimate partners through pherines, in addition to a well described olfactory pathway through odorants. Pherines are chemicals that have no odor but stimulate a specific epithelium in the nose (Monti-Bloch et al., 1994). In addition to influencing the choice of mate in regard to MHC status, there is robust literature (Cobey et al., 2012; Welling et al., 2012) in evolutionary psychology demonstrating that hormonal contraceptives affect the behaviors of both female and male partners which can cause changes in mate retention behaviors. These changes in mate retention behaviors could lead to intimate partner violence and death (Kaighobadi et al., 2009).

There is also suggestive evidence that hormonal contraceptives have the potential to modify brain structure and function. For example, some studies have implicated the reduction in the volume of the amygdala with depression associated with hormonal contraception (Lisofsky et al., 2016), as well as a study that reported a reduced volume of the hypothalamus (Chen et al., 2019), which could be related to the increased risk of suicide and suicide attempts in users (Skovlund et al., 2018). Formulations of hormonal contraceptives which have the greatest estrogen levels have been noted to have the greatest impact on the behaviors observed (Welling, 2013).

Hormonal contraceptives have also been found to be related to several factors that have the potential to influence relationships. Such factors include an increase in the expression of borderline personality disorder traits which could also add negative stressors to relationships, increase the risk of suicide and increase accidental deaths through substance abuse (Desoto et al., 2003).

Various psychosocial behaviors, such as jealousy, may be influenced by hormonal contraception and correlate with estrogen levels as well (Geary et al., 2001). Jealousy drives mate retention behaviors in both the male and female which can lead to intimate partner violence (Welling et al., 2012). Additionally, given that hormonal contraception does not always prevent pregnancy [Trussell, 2011; World Health Organization Department of Reproductive Health and Research (WHO/RHR) and Johns Hopkins Bloomberg School of Public Health/Center for Communication Programs (CCP) (2011)], its failure may lead to a termination of an unplanned pregnancy (i.e., an induced abortion; Bearak et al., 2020), which has been associated with some adverse mental outcomes in several epidemiological studies (Gissler and Hemminki, 1999; Fergusson et al., 2006; Coleman, 2011), some of which could result in death, such as suicide. Although it is not certain whether the epidemiological association between induced abortion and suicide is cause and effect or mediated by other factors, recent experimental data from an animal model provides strong biological plausibility that drug-induced pregnancy termination may influence adverse behavioral outcomes (Camilleri et al., 2019). Moreover, the literature also indicates decreased fertility among MHC similar partners (Beydoun and Saftlas, 2005; Meuleman et al., 2015). In addition to the potential effects of hormonal contraceptives on women and their relationships, these hormones may also impact the health of children conceived by women taking oral contraceptives, including the risk of pre-term delivery, low birth weight (Chen et al., 2009), and poor general health (Birnbaum et al., 2016). There is also experimental evidence in animal models supporting a potential mechanistic link to autism, discussed further below (Strifert, 2014; Zou et al., 2017; Li et al., 2018).

In the United States today, roughly 12 million women presently use hormonal contraceptives including the 6.4 million women who take oral contraceptives (estrogen-progestin combination drugs), known in common parlance as “the Pill” (Daniels and Abma, 2020). Given the widespread use of hormonal contraceptives, it is important to examine the potential links between hormonal contraceptive use and adverse medical outcomes.

## Choice of Intimate Partner, Major Histocompatibility Genes and Hormonal Contraception

As discussed below, some studies have demonstrated interesting and compelling evidence that hormonal contraception may influence a woman’s choice of mate by making her more likely to choose a male that is similar to herself in the major histocompatibility (MHC) genes. On the contrary, without the use of hormonal contraception, women are more likely to choose MHC dissimilar mates which is advantageous to the survival of their offspring. Scientists working in this field of study differentiate short- and long-term relationships. Various factors can influence those two relationship mate choices. There remains controversy as to whether or not these MHC preferences are a determining factor in choice of long-term and marriage relationships.

In humans, the MHC genes are also referred to as human leukocyte antigen (HLA) genes. The MHC genes provide immunological responses, such as combating infections, that impact survival of any child conceived during the relationship. The greater the number of dissimilar/different MHC alleles inherited, the greater the immunocompetence and the more likely a child might be able to summon a successful immune response to a greater number of pathogens, such as bacteria, viruses, or parasites, in order to survive. Before the advent of antibiotics in the 1940s, Western medicine was without effective pharmaceutical treatments for infections caused by bacteria and parasites. In the U.S., the average life expectancy in the 20th century before the development of antibiotics was 47 years with communicable diseases, a major cause of death. Life expectancy rose to 78.8 years after antibiotics became widely developed (Adedeji, 2016). A woman choosing a mate dissimilar to herself in the MHC genes could give her child a survival advantage. In 1900, 30% of all deaths in the United States occurred in children less than 5 years of age compared to just 1.4% in 1999. Infant mortality dropped from approximately 100 deaths per 1,000 live births in 1915 to 29.2 deaths per 1,000 births in 1950 and 7.1 per 1,000 in 1999 (Institute of Medicine, 2003). A recent study showed that offspring of women taking hormonal contraceptives were more infection prone, required more doctor visits, and suffered from more common illnesses (Birnbaum et al., 2016).

Moreover, the literature also indicates additional effects of MHC similarity on various factors that have the potential to negatively influence (e.g., increased stress) a relationship. Specifically, as mentioned above, there is decreased fertility among MHC similar partners (Beydoun and Saftlas, 2005; Meuleman et al., 2015). Additionally, offspring of MHC similar couples have an increased risk of autism and are more sickly (Strifert, 2014; Birnbaum et al., 2016). Associations between autism spectrum disorder and women’s oral contraceptive use have been found (Strifert, 2014; Li et al., 2018). There is also experimental evidence linking hormonal contraception to autism. Zou et al. (2017) showed prenatal exposure to levonorgestrel induces autism-like behavior in rats through ER beta suppression in the amygdala. Li et al. (2018) showed progestin used to prevent threatened abortion, progestin used at time of conception, and progestin contaminated seafood eaten during the first-trimester gestation resulted in autism in children. Also, pregnant rats eating fish fed endogenous progesterone or progestin had offspring with autism-like behaviors. Both fish and rats had ER beta receptor suppression in the brain. This experimental evidence concerning the amygdala is supported further by studies concerning hormonal contraception and brain structure and function, as discussed below. The stresses associated with caring for a sickly or autistic child can negatively impact parental relationships (Hartley et al., 2010; Lawrence, 2012). Offspring of MHC similar couples also have a higher risk of preterm birth and low/very low birth weight if mothers took contraceptives within 30 days prior to her last menstrual period, i.e., around the time of conception, again impacting the health of her offspring (Chen et al., 2009). Chen et al. (2009) proposed three possible mechanisms by which oral contraceptives near the time of conception can lead to low birth weight and preterm birth: (1) there are animal studies cited that support that high levels of estrogen caused fetal growth suppression; (2) the unexposed group is more likely to have had planned pregnancies and that pregnancies that are unplanned had been found by a previous study to be associated with low birth weight; and (3) women with unplanned pregnancies may be less compliant with the contraceptive regimen or more fertile. However, it should be noted that unplanned does not necessarily mean that the pregnancies were also unwanted, as all women in the study had Federal Canadian medical insurance and abortions are legal and widely available to Canadian women.

In addition to the influence MHC genes have on survival and longevity (Adedeji, 2016), the degree of similarity and dissimilarity of MHC genes between mates can influence their sexual relationships and behaviors. These behaviors, such as a female’s increase in extra-pair sexual partners and attraction to other men (Welling, 2013), would drive sexual jealousy and mate retention behaviors in the male (Kaighobadi et al., 2009), possibly leading to intimate partner violence, including death, as will be discussed further below.

In 1976, Yamazaki et al. (1976) discovered that mice preferred MHC dissimilar mates and used odor-mediated signals. Subsequently, birds, reptiles, and fish were also found to prefer MHC dissimilar mates. The MHC loci which had been found in mice, birds, fish, and humans are thought to help individuals choose genetically compatible mates, avoid inbreeding and increase heterozygosity and immunocompetence of offspring. In 1995, Wedekind et al. (1995) found women who had normal menstrual cycles (i.e., not taking hormonal contraceptives) preferred axillary odors of MHC dissimilar men. However, women taking hormonal contraceptives preferred the odors of MHC similar men.

In 2008, Roberts et al. (2008) investigated whether an individual woman’s odor preferences for MHC similar and dissimilar men would change whether she was taking hormonal contraceptives or not. He found that after starting hormonal contraceptives, women preferred MHC similar mates. He also found preference depended upon whether a woman was single or in a relationship. Single women who were not in a relationship preferred MHC similar men, while those in a relationship preferred MHC dissimilar men. The results regarding women’s odor preferences for MHC similar men while on hormonal contraceptives had been found in three previous studies (Wedekind et al., 1995; Wedekind and Furi, 1997; Santos et al., 2005), while two other studies showed an intermediate (Jacob et al., 2002) and a null effect (Thornhill, 2003).

In 2006, a study (Garver-Apgar et al., 2006) was published on romantic heterosexual couples with ovulating females. Both members were tested for alleles at three MHC loci. The researchers found that “as the proportion of MHC alleles couple shared increased, women’s sexual responsivity to their partners decreased, their number of extra-pair sexual partners increased, and their attraction to men other than their primary partners increased, particularly during the fertile phase of their cycles.” As the fertile phase of the cycle has higher levels of estrogen than the non-fertile phase, these effects on attraction and sexual responsivity may also be moderated by estrogen exposure. This is perhaps also the reason that hormonal birth control impacted MHC mate choice.

When studying MHC similarity/dissimilarity in long-term relationship couples, the evidence is very mixed for whether or not it is a consistent or an important factor. In a recent study (Saphire-Bernstein et al., 2017), “there was no effect of MHC similarity on any of the dependent variables used to measure in-pair attraction of 168 couples, but there were strong and consistent effects of MHC similarity on these measures in couples with two Asian partners (*N* couples = 44). In sum, our findings are consistent with an effect of MHC similarity on in-pair attraction within existing relationships, but they also suggest that this effect may be moderated by additional factors, particularly the ancestral background of the individual relationship partners.” Asian couples were the largest ethnic group representing 39% of their study cohort. A study of over 3,500 married couples (Croy et al., 2020) showed no MHC effect in married couples, although dissimilarity resulted in a low chance for homozygous offspring. Another study of married couples regarding MHC similarity/dissimilarity (Chaix et al., 2008) showed sampled European Americans to have statistically significantly favored MHC-dissimilar mates, while African couples did not, but without a statistical significance.

While there is a possibility that various factors (e.g., the development of antibiotics, intellectual abilities) may also impact long-term mate choices, in addition to MHC similarity/dissimilarity, there is evidence that hormonal contraceptives have an impact on mate choice and aspects of sexual relationships by changing responses of women on hormonal birth control to compounds derived from male axillae when compared to naturally cycling women. A 2012 study (Roberts et al., 2012) found that although women who used hormonal contraception scored lower on measures of sexual satisfaction and partner attractiveness, they were more satisfied with their partner’s provision and so they had longer relationships and were less likely to separate than women who were not using hormonal contraception when they partnered. However, it may be only short-term relationships are impacted.

In many MHC studies, it is believed that it was the odor of compounds in the secretions of men’s axillae that caused women to respond and that may be true in many or most circumstances. Surrounded by much controversy, there is also evidence that compounds that are not perceived as odors or that can stimulate the olfactory cells of the nose can also affect another part of the nose, the vomeronasal organ, resulting in behavioral changes in women.

## Pheromones, Odorants, and The Human Vomeronasal Organ (VNO)

There is controversy in the literature (Meredith, 2001; D’aniello et al., 2017; Stoyanov et al., 2018; Gebhart et al., 2019) in regard to whether or not there is a functioning vomeronasal organ (VNO) in humans. The VNO is the organ that perceives pherines or pheromones influencing reproductive behaviors in non-humans. While recognized as a discrete organ in the human fetus (Marini et al., 2019), it is considered a vestigial organ in humans by some scientists (Smith et al., 2014). Previous literature (e.g., Stoyanov et al., 2018) has described its anatomical location on the nasal septum approximately 2 cm from the nares with a small punctuate 1 mm opening into a small diverticulum makes it difficult to locate without instrumentation in live humans. The following will not resolve the controversy (nor is it meant to) but will supply information that is not often discussed in the literature regarding the existence of a functioning human VNO which is able to distinguish different compounds and provide compelling physiologic evidence at the cellular level.

In 1994, Monti-Bloch et al. (1994) published a study testing the respiratory (RE), olfactory (OE), and vomeronasal (VNO) epithelia of the human nose using a 0.3 mm electrode placed directly on the epithelium. Electrophysiological recordings (EVGs) were made of the responses of 30 males and 30 females. A mini probe was used, constructed of concentric Teflon tubes around an electrode stabilized by a nasal retractor. The internal tube diameter was 1 mm and used to precisely deliver a stimulant while an external 2 mm tube was used to scavenge (remove after stimulation) the stimulant. This prevented the stimulant from acting on odor or respiratory epithelium. They were able to demonstrate that a putative pheromone, or pherine, as they named it, would stimulate the VNO, creating an electrogram, but not stimulate the OE or RE of the same subject. Similarly, an olfactant created an electrogram when pulsed on the OE, but not in the VNO or on the RE epithelia. They also demonstrated varying EVG responses in response to distance from stimulus placement and variance with the concentration/strength of the stimulus. The VNO stimulants (pheromones/pherines) also produced sexually dimorphic responses. The results of the stimulation of the VNO epithelia resulted in several types of responses of the autonomic nervous system causing decreased skin resistance and an increase in skin temperature. In the central nervous system, there was increased alpha-cortical activity. The subjects were not able to detect an odor of the putative pheromones or pherines which stimulated the VNO. One of the chemical/pherines, PH94B, studied by Monti-Bloch et al. (1994), was used in a phase 2 randomized, double-blind, placebo-controlled study. This pherine was successfully tested as an intranasal aerosol to treat social and performance anxiety in women. The positive result was reported upon in 2014 (Liebowitz et al., 2014).

In 1998, Monti-Bloch et al. (1998a) also demonstrated that stimulation of the male VNO with pregna-4,20-diene-3,6-dione (PDD) resulted in EVGs and reductions of serum testosterone and LH (luteinizing hormone). There was also a reduction in heart rate due to increased vagal tone (parasympathetic activity) and a decrease in respiration.

A 1998 review, “The Human Vomeronasal System”, (Monti-Bloch et al., 1998b) reported upon functional brain imaging studies with consistent activation of the hypothalamus, amygdala, and cingulate gyrus-related structures with stimulation of the human VNO.

In 2009, Czech Republic researchers reported their observational hypothesis (Foltan and Sedy, 2009) regarding patients whose orthognathic surgery (surgery which corrects congenital or acquired jaw deformities) often requires maxillary osteotomy which results in the destruction of the VNO. A previous study of orthognathic patients had shown that 24% of patients reported an improvement in the formation of heterosexual relationships. Patients also reported a 24% improvement in their social lives even though preoperatively they were unaware that there had been anything lacking. Despite being psychologically normal preoperatively, the authors’ experience with more than 1,000 patients who had undergone orthognathic surgery noted that their social relationships regarding their mates resulted in frequent mate change and the development of promiscuity. These responses differed from the responses of other cosmetic surgery patients who also had enhancements of their appearances, i.e., the authors could not explain their observations of the orthognathic patients solely upon an enhanced appearance. The authors’ presumption was that the VNO had an inhibitory role in the identification of inappropriate individuals for mating which ended with the destruction of the VNO during surgery. They concluded a comprehensive study should be done regarding these observations of orthognathic patients in order to test their hypothesis of VNO loss through surgery with resultant loss of negative feedback for exclusion of inappropriate mates.

In the 2001 review article of the evidence from the literature both supporting and not supporting a functional human VNO, the author stated, “The EVG is the best evidence for a selective chemosensory process in the VNO region. Systemic responses to restricted VNO region stimulation are an important stumbling block for the hypothesis that there is no special chemosensitivity in this region.” (Meredith, 2001). The review also stated that EVG recordings of the potentials of stimulated cells should not be disregarded.

It should also be recognized that in addition to the VNO, other pathways through the olfactory and accessory vomeronasal olfactory pathways are involved in behaviors of animals and humans mediated by pheromones (Baum and Cherry, 2015; Cherry and Baum, 2020). Both pathways, olfactory and VNO, may be operative in humans. There is also great specificity in terms of structural compounds that will stimulate the VNO. For instance, in humans, androst-4-en-3-one is active upon the VNO, but 5a-androst-16-en-3-one is not, although it is very closely chemically related.

While the previously mentioned literature describes the potential for a functioning VNO in humans, there is also robust literature which provides evidence that the human VNO is a non-functioning vestigial organ. The family of vomeronasal receptor type-1 (V1R) genes encodes pheromone receptors. A 2018 study by Suzuki et al. (2018) found that the loss of the ancient V1R gene “…in some tetrapods is coincident with the degeneration of the vomeronasal organ in higher primates, cetaceans, and some reptiles…”. It has also been shown that humans lack an accessory olfactory system necessary for the functioning of the VNO, which is present in other mammals (Smith and Bhatnagar, 2019), as well as vomeronasal nerves (Salazar et al., 2019). Despite the presence of the VNO and accessory olfactory bulbs demonstrated in human fetuses, these organs may later undergo involution. Additionally, a 2015 review article (Brennan and Keverne, 2015) of the chemosensory systems, some of which involve pheromones, noted that “In primate mammals, the gene pool for olfactory receptors has diminished while most of the vomeronasal receptor genes and their specific ion channels are non-functional pseudogenes.” The authors interpret this as an indication that these diminished with the evolution of the neocortex which uses primarily auditory and visual information for decision making.

However, as described above, the literature which demonstrates that a discrete human organ, the VNO, can be differentially stimulated by chemicals that are not odorants and can result in changed behaviors and emotions, supports the presumption of a functioning VNO in humans. Also, clinical observations of patients in which the VNO is impacted supports its role in human behavior (Foltan and Sedy, 2009). It is the VNO pathway to the hypothalamus that may account for physiologic changes noted over the last 50 years that are otherwise inexplicable, such as the greater attraction and preference for MHC dissimilar mates of women with normal fertile menstrual cycles (Roberts et al., 2008). The development of a therapeutic drug that is known through the study of stimulating the human VNO, and which resulted in a rapid diminution of social anxiety symptoms in women, demonstrates a connection between the VNO and the central brain regulating mood (Liebowitz et al., 2014). However, it should be stressed that regardless of whether a functioning VNO exists or not, there are organic chemical compounds that initiate and influence human behaviors, especially those involved in human reproductive behaviors. Through the sensory stimulation of the VNO and olfactory organ, the perception of MHC similarity/dissimilarity can be influenced by whether or not a woman is cycling naturally or by hormonal contraception.

## Hormonal Contraception, Brain Structure and Function, and Mood Disorders

Hormonal contraceptives may have been found to modify both structure and the functioning of various brain structures. The amygdala plays an important role in emotion and behavior, the hypothalamus regulates mood, menstrual cycles, libido, appetite, sleep cycles, heart rate, and temperature, and the hypothalamus connects the endocrine to the nervous system. There are two types of estrogen receptors, alpha, and beta, located in the brain which regulate the reproductive system, as well as mood, and emotion. In the brain, alpha estrogen receptors play a critical role in regulating reproductive neuroendocrine behavior and function. These receptors mediate the impact of potent synthetic estrogens found in hormonal contraception on behaviors and possibly brain structure, described below. The limbic system in the forebrain controls mood and emotion and is rich in beta estrogen receptors. The amygdala and hypothalamus are part of the limbic system.

In relation to the effect of hormonal contraception on brain structure, in 2020, an article was published, which reviewed 33 articles, 23 of which were functional investigations and 10 were structural studies (Bronnick et al., 2020), which included a study showing reduced gray matter volume of the left amygdala (Lisofsky et al., 2016).

Included in the functional studies was a randomized controlled trial (Merz et al., 2012) which found that women on oral contraceptives had enhanced fear learning when exposed to cortisol and electrical stimulation, perhaps resulting in increased anxiety in women taking hormonal contraceptives. Cognitive and emotional processes potentially underlying PTSD (post-traumatic stress disorder) symptoms were found to be heightened by hormonal contraception in the insula and dorsal anterior cingulate cortex (both involved in the fear processing network of the brain) during traumatic film viewing (Miedl et al., 2018). Hormonal contraception has also been found to interfere with oxytocin, a peptide and key modulator of pair-bonding in men and women. Women given oxytocin had increased perceived attractiveness of their partner compared to other men with elevated response in their nucleus accumbens, a reward associated region. This has been previously found to be true in men as well. However, women taking hormonal contraception had no enhanced response in the nucleus accumbens; their response to oxytocin was absent (Scheele et al., 2016). These studies reveal that hormonal contraception has effects upon other important hormones, such as cortisol and oxytocin, that can impact a woman’s emotions and ability to pair-bond.

A study of the amygdalae in adolescents with bipolar disorder (BD) revealed “Decreased volume and increased response of emotional stimuli in BD adolescents are consistent with previous reports” (Kalmar et al., 2009). The authors also reported their data supported a structure-function relationship, noting an inverse relationship between volume and response to emotional stimuli, suggesting a “possible pathophysiological link” in BD patients. A reduction in the size of the amygdala in women using hormonal contraceptives had been found in previous studies (Lisofsky et al., 2016). A study of depressed and never-depressed individuals found that depressed individuals had smaller left amygdalae (Siegle et al., 2003). They also found sustained functional activity to negative vs. positive words. Moreover, in addition to a decline in mood, women using combined oral contraceptives were found to experience changes in the reactivity of the left insula and right amygdala, both areas of the brain associated with emotion (Gingnell et al., 2013).

In 2019, an abstract was presented at the December 4, 2019 meeting of the Radiological Society of North America by Ke Xun Chen titled “Oral contraceptive use is associated with smaller hypothalamic volumes in healthy women”. The study found a statistically significant decrease in the MRI measured volume (size) of the hypothalamus in women who took hormonal contraception. They also found a significant correlation between a smaller hypothalamus and greater anger and a strong correlation with depression. They found no correlation with cognitive testing results. This study showed that there were both structural and functional changes in the brain with hormonal contraceptive use (Chen et al., 2019). Although the smaller volume of the hypothalamus only suggests that size is related to function as found with the amygdalae, a study (Holle et al., 2011) found that size reduction of the posterior hypothalamus is related to hypnic headaches, a rare disorder that causes nighttime headaches. It must be noted that this abstract (Chen et al., 2019) represents a small singular study of the hypothalamus with only 21 of the 50 participants taking hormonal contraceptives and will need subsequent confirmatory research before any conclusions can be drawn.

It is known that neural systems implicated in reactive aggression (amygdala, hypothalamus, and periaqueductal gray; the basic threat system) are critically implicated in anger (Blair, 2012). The high levels of estrogen in hormonal contraceptives may just be temporarily suppressing a portion of its function resulting in some atrophy, such as that which occurs in muscle tissue with lack of exercise. More research is needed to further explore this concept. However, in regards to the emotional effect of increased depression, this study supports the association of hormonal contraceptives and depression, suicide, and anger.

In April 2018, Danish researchers published a study of nearly half a million women followed over 8 years which found that women who took hormonal contraceptives had a statistically significant increase of 97% in suicide attempts and 208% in suicide (Skovlund et al., 2018). There was a change in effect just 2 months after the initiation of hormonal contraceptives, which is supportive of the findings of Lisofsky et al. (2016) which noted changes at 3 months after exposure to hormonal contraceptives. The authors also noted the different risks for suicide attempts associated with different formulations. In concordance with a 2012 study correlating behavioral changes with estrogen levels (Welling et al., 2012), this author noted that in the Skovlund et al. (2018) study, the hormonal formulations and the known estrogen levels of those formulations also indicated that the higher the level of estrogen in the formulation, the higher the risk of the first suicide attempt.

There is also evidence that the age when hormonal contraception is first started impacts the likelihood of major depressive disorder (MDD). Women who started hormonal contraception while still adolescents had long-term increase in MDD regardless of current use. It is postulated that there are changes that occur which are more lasting if the hormonal contraception is used during the final brain maturation before adulthood (Anderl et al., 2020).

Additionally, in 2003, researchers found that women with borderline personality disorder (BPD) had their symptoms become markedly worse when starting hormonal contraception (Desoto et al., 2003). They also found that symptoms varied with estrogen levels during a normal fertile menstrual cycle with worsening of symptoms when estrogen levels were highest (Desoto et al., 2003). During the natural menstrual cycle, there are marked changes in levels of estrogen, the highest level occurring just prior to ovulation. In patients with BPD, behaviors can include: a pattern of unstable intense relationships, impulsive and risky behavior, such as reckless driving, unsafe sex, drug abuse, or ending a positive relationship, suicidal threats or behavior or self-injury, wide mood swings lasting from a few hours to a few days, which can include intense happiness, irritability, shame or anxiety, and intense anger, such as frequent loss of temper and having physical fights (National Institute of Mental Health). Clearly, these behaviors would increase the risk of violent death in those suffering from BPD (National Institute of Mental Health).

Borderline personality disorder does not appear to have a known causal relationship with hormonal contraception. However, hormonal contraception can increase the behaviors of BPD that can be life-threatening, such as risk-taking and drug abuse, which can lead to accidental death. As borderline personality disorder is the most common personality disorder with a prevalence of 10% of all psychiatric outpatients and between 15 and 25% of inpatients (Lieb et al., 2004; Leichsenring et al., 2011), many women are at risk and can be impacted. In the U.S., the prevalence of BPD is 1.8% and 70% are women (Lieb et al., 2004). With a U.S. population of 328.8 million, there are approximately 4.1 million women with BPD. With 14% of the 61 million women aged 15–44 using oral contraceptives in the U.S. (Centers for Disease Control and Prevention, 2020), there are approximately 8.5 million women at risk if they have a diagnosis of BPD. This can be a significant public health risk.

## Hormonal Contraception and Psychosocial Behaviors

In 2002, researchers at the University of New Mexico studied the changes in women’s and their partners’ sexual and mate retention behaviors as they related to their menstrual cycles (Gangestad et al., 2002). Care was taken that all subjects were fertile as evidenced by detecting ovulation. A standardized questionnaire regarding mate retention behaviors was used. They found that when women were in the fertile phase of their cycle, they were more interested in sexual relations with a man that was not their partner than their own partner. Women also were found to initiate sexual relations more frequently when fertile. Male partners were found to have increased vigilance tactics in their mate retention behaviors around the time of ovulation as observed by their partners (Gangestad et al., 2002). As women are only fertile 4–5 days a month, these preferences would be important to increase the probability of conception.

In 2007, these preferences for women in their fertile phase were confirmed by another University of New Mexico study (Miller et al., 2007) which found that there was a connection between the menstrual cycles of female lap dancers and the tips they earned during a given night. Women who were taking hormonal contraception earned $193 per shift compared to non-users who made $276 per shift. Women in their fertile phase made higher tips than when they were not fertile. Women who were not fertile had no variation in tips received (Miller et al., 2007).

In a 2012 study, Welling et al. (2012) found that women using hormonal contraception reported, “more intense affective responses to partner infidelity and greater overall sexual jealousy than women not using hormonal contraceptives”. This resulted in increased mate retention tactics of not only the female but also of the male. The degree of retention behaviors in the female correlated with the dose of estrogen, but not progestin, in the hormonal contraception taken. These mate retention behaviors were not only self-reported but also correlated with their mate’s perception of their behaviors.

Additional research Cobey et al. (2012) examined the differences in the levels of self-reported jealousy and found they varied not only with the menstrual cycle but with whether women were single or partnered. Single women reported levels of jealousy between fertile and infertile cycle phases and were not significantly different from either. Partnered women had levels of jealousy which were significantly higher than the non-fertile phase and similar to the level found when fertile. Partnered women may be more likely to have offspring and need the resources provided by their mate. Women on hormonal contraceptives also used mate retention tactics more frequently. Most likely, this behavior resulted from the more intense and greater sexual jealousy that women on hormonal contraceptives feel as indicated by self-report measures (Geary et al., 2001).

In 2013, a lengthy review article was published examining the psychobehavioral effects of hormonal contraception on both humans and primates given contraceptives to control captive populations (Welling, 2013). In summary, the findings were that “women who used hormonal contraception reported higher rates of depression, reduced sexual functioning, and higher interest in short-term sexual relationships than their naturally-cycling counterparts.” (Welling, 2013). There was also evidence that hormonal contraception decreased the ability to attract a mate and increased mate retention behaviors of both partners. Evidence indicated that hormonal contraception altered mate choice and negatively affected sexual satisfaction in parous women (Welling, 2013).

The best evidence available today suggests that hormonal contraception has the potential to adversely affect relationships. Sexual jealousy influenced by hormonal contraceptives may lead to mate retention tactics that can include intimate partner violence (IPV). As found by Welling et al. (2012) and Welling (2013), sexual jealousy is increased in both the woman and her partner, and there was higher interest in short-term sexual relationships while using hormonal contraceptives. Cobey et al. (2012) found partnered women had significantly higher levels of jealousy while taking hormonal contraceptives. A New Zealand study found that “Women who had experienced IPV were significantly more likely to have ever used contraception.” (Fanslow et al., 2008). Intimate partner violence may be one mechanism by which hormonal contraception’s influence on increased sexual jealousy leads to an increased risk of violent death. The tendency for greater numbers of sexual partners among users creates relationship instability and sexual jealousy, although it is not known if hormonal contraception causes women to desire more sexual partners or if they take hormonal contraception because of their natural proclivities.

Sexual jealousy is the driver of mate retention tactics which can include vigilance, as well as threats of violence and violence against a mate. Anger develops when there is a threat of a loss, such as that of a mate, resulting in sexual jealousy and mate retention behaviors some of which can lead to intimate partner violence and death.

## Mate Retention Tactics and Mate Retention Inventory

In 1988, the Mate Retention Inventory (MRI) was developed to standardize the measurement of mate retention tactics for use in psychological studies. An inventory of 19 mate retention tactics, posing several questions per tactic, resulted in 104 behavioral questions which study participants would answer for a score in the MRI (Buss, 1988).

In 2008, a short form of the Mate Retention Inventory was developed to make its use in psychological studies more practicable by shortening the number of questions in the inventory (Buss et al., 2008). The authors found a high correlation with the longer inventory of 104 behaviors of the specified 19 tactics when just 38 behaviors were used. The mate retention tactics of vigilance, monopolization of mate’s time, emotional manipulation, and derogation of competitors were shown to be correlated with partner directed violence. For example, Vigilance: “Called to make sure my partner was where she said she would be” and “Snooped through my partner’s personal belongings”; Emotional Manipulation: “Pleaded that I could not live without my partner” and “Told my partner that I was dependent on my partner”; Monopolization of Time: “Insisted that my partner spend all her free time with me” and “Spent all my free time with my partner so that she could not meet anyone else”.

Mate retention tactics can be caused by sexual jealousy. Some tactics are benign and pose no threat, such as enhancing one’s appearance or going to a nice restaurant. Other tactics have been found to lead to intimate partner violence which can be emotional, verbal, or physical in nature (Buss, 1988; Buss et al., 2008). Tactics such as vigilance, monopolization of time, and emotional manipulation might be early signs to predict future physical violence so that possible interventions could be implemented to protect and inform women. Unfortunately, intimate partner physical violence is reinforced behavior as it is a successful tactic for the male to maintain the relationship.

In a 2009 review, “From Mate Retention to Murder: Evolutionary Psychological Perspectives on Men’s Partner-Directed Violence” (Kaighobadi et al., 2009), the authors reviewed the literature regarding predictors of female-directed violence. They stated, “The problem of paternity uncertainty is hypothesized to have selected for the emotion of male sexual jealousy, which in turn motivates men’s nonviolent and violent mate retention behaviors. We review empirical evidence for the relationships among paternity uncertainty, male sexual jealousy, and men’s partner-directed violence.” (Kaighobadi et al., 2009). As described above, hormonal contraception has been found to increase interest in short-term sexual relationships which could lead to paternity uncertainty and increased jealousy. These factors may have contributed to the finding of increased violent death rates in ever-users of hormonal contraceptives.

## Hormonal Contraception Failure, Unplanned Pregnancy, and Induced Abortion

In 2012, Eisenberg et al. (2012) conducted a study that demonstrated that 45% of women overestimated the effectiveness of hormonal contraception. Confident in their efficacy, women using hormonal contraception did not realize that there was a 9% chance of pregnancy occurring while taking oral contraceptives as directed (Centers for Disease Control and Prevention, 2021). As users of hormonal contraception are largely unaware of this well-documented failure to prevent pregnancy, this can lead to unintended and unplanned pregnancies. In countries where induced abortion is legal, the unplanned pregnancy may be aborted. Some of the users of hormonal contraception in the Hannaford et al. (2010) and Charlton et al. (2014) cohorts may have had an unplanned pregnancy due to failure of their hormonal contraception to prevent pregnancy. Some of these pregnancies may have resulted in abortion. There are studies (Gissler et al., 1996; Fergusson et al., 2006, 2013; Coleman, 2011) which have correlated adverse mental health outcomes with abortion, some of which may potentially be linked to violent death. It cannot be excluded that induced abortion may have been a confounding variable in the Hannaford and Charlton cohorts.

Due to large numbers of women using hormonal contraception who may have abortions, there may be adverse public health consequences. According to the Centers for Disease Control and Prevention (CDC), oral contraceptives have a 9% pregnancy rate with typical use (Centers for Disease Control and Prevention, 2021). Typical use is understood to mean that over the course of a year, having sexual intercourse and using birth control every time, but possibly not following the directions for exactly how to use it correctly. There are approximately 6.4 million women taking oral contraceptives or 14% of the 72 million women aged 15–49 (Daniels and Abma, 2020). This results in 576,000 unplanned pregnancies a year. According to the Guttmacher Institute (2019), 42% of women with unplanned pregnancies choose induced abortion, resulting in 241,000 abortions a year. More than half (54%) of women seeking abortions had used a method of contraception the month they became pregnant and of these 14% reported using oral hormonal contraception, as well as other forms of hormonal contraception (Jones, 2018).

In 2011, Coleman performed a meta-analysis of 22 studies concerning the association of induced abortion and adverse mental health outcomes compared to women without abortion, delivery of unintended pregnancy, and delivery of an intended pregnancy (Coleman, 2011). She found increased population attributable risks of 8.5% for depression, 10.7% for alcohol, 26.5% for marijuana use, and 20.9% for all suicidal behaviors. Overall, there was an 81% increased risk of mental health problems post-abortion (Coleman, 2011). The meta-analysis showed a population-attributable risk (PAR) percentage of 34% for suicide as an outcome. This meta-analysis sparked controversy in the literature, especially as its findings were contrary to those of a recent review from the Royal College of Psychiatrists (National Collaborating Centre for Mental Health at the Royal College of Psychiatrists, 2011). One critical analysis was published the following year (Steinberg et al., 2012), enumerating several flaws in the Coleman (2011) meta-analysis, which were reported to make the meta-analysis conclusions invalid. These flaws were largely methodological in nature. For example, Coleman was said to have violated guidelines for conducting meta-analyses, such as unclear exclusion criteria for studies and a conflict of interest as Coleman was the author of 11 of the 22 studies in the meta-analysis. Other criticisms were that out of 22 studies, only 14 different data sets were used, causation was assumed in calculating population attributable risk and the comparison group was misclassified, i.e., instead of using women who delivered an unplanned pregnancy as a control group, the comparison should have been women without a pregnancy. In addition to the findings of Coleman’s meta-analysis, a prospective longitudinal study in New Zealand (Fergusson et al., 2006) found that major depression, suicidal ideation, illicit drug dependence and a number of mental health problems were statistically significantly increased in post-abortive women. A Finnish record linkage study (Gissler et al., 1996) found that the suicide rate post-abortion was three times the general suicide rate and six times the rate associated with giving birth.

In 1999, a record-based Finnish study (Gissler and Hemminki, 1999) of deaths among 15–49-year-old women from 1987 to 1994 found one-third of all deaths were violent. Up to 1 year after induced abortion, there was a statistically significant increase of 340% in homicides, 273% in suicides, and 111% in accidents (Gissler and Hemminki, 1999). Due to the increased suicide rate after induced abortion, abortion post-care guidelines were changed to include a visit where women were screened. As Gissler et al. (2014, 2015) previously reported, women were evaluated 2–3 weeks after the abortion for mental health disorders. Subsequently, the excess suicide rate declined from over three-fold to over two-fold. This is important to consider as substance abuse and suicidal behaviors can lead to accidental and violent deaths.

Several studies have also documented that a leading cause of death of pregnant women is homicide, usually by an intimate partner (Maryland Department of Health and Mental Hygiene, 2013; Wallace et al., 2020). Thirty % of U.S. female homicides are perpetrated by an intimate partner (Catalano, 2007).

## Conclusion

This review provides compelling research of the possible multiple psychological and physiological factors that may contribute to the findings of two large prospective cohort studies which found statistically significant correlations of hormonal contraception with a higher risk of violent death by suicide, accident, or homicide. It also seeks to provide clinicians prescribing and caring for patients using hormonal contraception with the information needed to assure their patients’ continued well-being.

The findings of this review support the need for screening women who are prescribed hormonal contraception and provide the information needed to obtain informed consent. Screening for depression, borderline personality disorder, and intimate partner violence could lead to a reduction in violent deaths. Women also need to be provided with documented adverse consequences of hormonal contraception to become cognizant of possible symptoms that could portend an adverse outcome. For instance, the onset of depressive symptoms might be better treated not by taking a prescribed antidepressant, but by changing from a newly prescribed hormonal form of contraception to one without hormones but equal efficacy.

Clinical recommendations are based upon what a reasonable person would want to know both legally and ethically under the standard of care guidelines for informed consent and must be followed when prescribing drugs or performing surgery. All severe risks (death, paralysis, loss of cognition, loss of a limb) should always be disclosed, even if the probability of occurrence is negligible (Paterick et al., 2008). Women taking hormonal contraceptives should be apprised of all the critical risks of taking hormonal contraception, including the potential for effects shown to be associated with an increased risk of violent death, in order to make an informed choice. Physicians should be educated about the risk of a violent death so that their patients can be screened for intimate partner violence, depression, suicide risk, and borderline personality disorder. Physicians are presently urged by professional societies to screen for intimate partner violence due to a high prevalence in U.S. society.

According to the Merriam-Webster dictionary, a definition of *contraindication* is “something (such as a symptom or condition) that makes a particular treatment or procedure inadvisable” (Merriam-Webster Dictionary, 2021). Relative contraindications call for an individualized assessment of the risks; if the benefit outweighs the risk, then the relative contraindications may be disregarded. It is this author’s opinion that patients with borderline personality disorder have a relative contraindication to using hormonal contraceptives (Desoto et al., 2003) and should be both apprised of the worsening of symptoms, i.e., depression, substance abuse, and risk-taking behavior, and screened appropriately. If a worsening of the BPD symptoms was observed, then an early psychiatric assessment and interventions may potentially be lifesaving, including the possible recommendation of non-hormonal methods of birth control.

Patients need to be aware that oral contraceptives, used as most women take them, still result in a 9% chance of becoming pregnant. Without feeling the stress and anxiety of an unexpected pregnancy, they may be able to more clearly reason whether the impact of birth, adoption, or an induced abortion would result in the best outcome for them. They could also discuss these options with their partner, as studies support both the mental harms and increased risk of death following abortion.

Homicide is a leading cause of death of pregnant women. Unplanned pregnancy due to imperfect fertility control, followed by induced abortion, is also a major risk for all forms of violent death: homicide, suicide, or accident. Patients should also be apprised of the impact of hormonal contraception on the health of their offspring and themselves.

There is also evidence that the detection of pherines/pheromones of males by females, potentially by the vomeronasal organ, affects the attraction of a mate by discerning MHC dissimilarity and similarity. However, this does not exclude the evidence that odorants also have an effect and that, in humans, both pathways, can be operating. Hormonal contraception was found by many studies, though not all studies, to change a female’s attraction toward mates that are similar, which results in women being less sexually responsive and more likely to have extra-pair bondings, resulting in sexual jealousy and anger in their mates. Hormonal contraception can increase feelings of jealousy in paired women and can incite more mate retention behaviors of both partners that can lead to intimate partner violence. The estrogen level in the formulation of hormonal contraceptives used correlates to both the degree of jealousy engendered, mate retention behaviors, and suicidal ideation. Hormonal contraception is also associated with structural brain changes of the amygdalae, as well as depression. One small study also revealed an effect on the size of the hypothalamus which also correlated with feelings of depression and anger compared to women not taking oral contraceptives. Suicide is also a major concern with all hormonal contraceptive use and correlates with estrogen content. Women who take hormonal contraception have an increased risk of suicide and accidental death, through the potential worsening of symptoms of borderline personality disorder, i.e., depression, substance abuse, and risk-taking behavior.

There is a great need for further research concerning brain function and structure relating to exposure to hormonal contraceptives, especially since these drugs are often given to young women whose brains are not fully matured. Through the use of physician screening and patient education, the incidence of violent death can potentially be mitigated. It cannot be denied that millions of women are potentially affected adversely by hormonal contraception and its potential link to increased suicide, intimate partner violence, induced abortion, and worsening of BPD traits, such as substance abuse and risk- taking. These women are likely represented in the findings of two large prospective cohort studies indicating that women taking hormonal contraception have an increased risk of violent death. Just as suicide rates declined when women post-abortion were screened (Gissler et al., 2014, 2015), through informed consent, education, and screening for the risks reported to be associated with hormonal contraception, it is proposed that many women’s lives could potentially be saved.

## Author Contributions

AL conducted the literature review, wrote the first draft of the manuscript, and edited and approved the final manuscript.

## Conflict of Interest

The author declares that the research was conducted in the absence of any commercial or financial relationships that could be construed as a potential conflict of interest.

## Publisher’s Note

All claims expressed in this article are solely those of the authors and do not necessarily represent those of their affiliated organizations, or those of the publisher, the editors and the reviewers. Any product that may be evaluated in this article, or claim that may be made by its manufacturer, is not guaranteed or endorsed by the publisher.
